# Bioprotective properties of Dragon's blood resin: *In vitro *evaluation of antioxidant activity and antimicrobial activity

**DOI:** 10.1186/1472-6882-11-13

**Published:** 2011-02-17

**Authors:** Deepika Gupta, Rajinder K Gupta

**Affiliations:** 1 University School of Biotechnology, GGS Indraprastha University, Sector 16C, Dwarka, New Delhi, 110075, India

## Abstract

**Background:**

Food preservation is basically done to preserve the natural characteristics and appearance of the food and to increase the shelf life of food. Food preservatives in use are natural, chemical and artificial. Keeping in mind the adverse effects of synthetic food preservatives, there is a need to identify natural food preservatives. The aims of this study were to evaluate *in vitro *antioxidant and antimicrobial activities of Dragon's blood resin obtained from *Dracaena cinnabari *Balf f., with a view to develop safer food preservatives.

**Methods:**

In this study, three solvents of varying polarity were used to extract and separate the medium and high polarity compounds from the non-polar compounds of the Dragon's blood resin. The extracts were evaluated for their antimicrobial activity against the food borne pathogens. The antioxidant activities of the extracts were assessed using DPPH and ABTS radical scavenging, FRAP, metal chelating and reducing power assays. Total phenolics, flavonoids and flavonols of extracts were also estimated using the standard methods.

**Results:**

Phytochemical analysis of extracts revealed high phenolic content in CH_2_Cl_2 _extract of resin. Free radical scavenging of CH_2_Cl_2 _extract was found to be highest which is in good correlation with its total phenolic content. All test microorganisms were also inhibited by CH_2_Cl_2 _extract.

**Conclusions:**

Our result provide evidence that CH_2_Cl_2 _extract is a potential source of natural antioxidant compounds and exhibited good inhibitory activity against various food borne pathogens. Thus, CH_2_Cl_2 _extract of Dragon's blood resin could be considered as possible source of food preservative.

## Background

There are many pathogenic organisms known to spoil refrigerated and ready to eat food products, often leading to food poisoning. Besides this all refrigerated and packed foods also undergo autooxidation during storage leading to formation of reactive oxygen species (ROS). These ROS cause oxidation of lipids producing secondary oxidants like heptanol and hexanal which lead to oxidative rancidity and deteriorate the flavor of the food [1,2]. Thus, preservatives may be antimicrobials, which inhibit the growth of bacteria or fungi, including mold, or antioxidants, which inhibit the oxidation of food constituents. Antioxidants also play an important role in preventing chronic diseases by reducing the oxidative damage to cellular components caused by ROS e.g. superoxide anions, hydrogen peroxide and hydroxyl, nitric oxide radicals. Hence, intervention of an antioxidant may have a therapeutic effect and also maintain the freshness of food products. A wide range of synthetic antimicrobial agents like sodium benzoate, sorbate and synthetic antioxidants like butylated hydroxy toluene (BHT), butylated hydroxyl anisole (BHA) are in use as food preservatives [3] but can cause liver damage, mutagenicity and neurotoxicity. Because of the lack of more safer and effective preservatives, lot of research is being directed towards searching the food preservatives of natural origin.

Dragon's blood is a name applied to many red resins described in the medical literature. *Dracaena *spp., *Daemonorops *spp., *Croton *spp. and *Pterocarpus *spp. are different sources of Dragon's blood [4,5]. Dragon's Blood resin in unani medicine is commonly known as Dammul-akhwain. It has wide medicinal uses: haemostatic, antidiarrhetic, antiulcer, antimicrobial, antiviral, wound healing, antitumor, anti-inflammatory and antioxidant [6,7]. There are few reports on antioxidative activity and chemoprotective potential of compounds isolated from resin [8,9]. The antimicrobial and antiviral potentials of resin extracts have previously been studied [10-13]. Previous studies have revealed that the resin is rich in flavanoids, homoisoflavanoids, chalcones, sterols and terpenoids [14-21]. Since it is the whole resin which is used in traditional medicinal systems, it is essential to have definite information on antioxidant potential of this resin. Being rich in phenolic compounds this resin holds possible use as food preservative, which has not been studied yet. This is the first ever report evaluating potential of dragon's blood resin as food preservative. The aim of this study was to evaluate *in vitro *antioxidant activity and antimicrobial activity of this resin to support its candidature as food preservative.

## Methods

### Chemicals

All chemicals and reagents used were of analytical grade and obtained mostly from Sigma. Folin-Ciocalteu phenol reagent, Gallic acid, Rutin, BHT, 6-Hydroxy-2,5,7,8-tetramethyl-chroman-2-carboxylic acid (Trolox), 2,20-Azinobis-3-ethylbenzothiazoline-6-sulphonic acid (ABTS), 2,4,6-Tripyridyl-2-triazine (TPTZ), 1,1-Diphenyl-2-picrylhydrazyl (DPPH), [4,40-[3-(2-Pyridinyl-1,2,4-triazine-5,6-diyl] bisbenzenesulfonic acid] (Ferrozine), Nitro blue tetrazolium (NBT), Nicotineamide adenine dinucleotide (NADH), Phenazine methosulphate (PMS), Griess reagent, 2-(4-Iodophenyl)-3-(4-nitrophenyl)-5-phenyltetrazolium chloride (INT), 3-(4,5-dimethyiazol-2-yl)-2,5-diphenyltetrazolium bromide (MTT) and Trichloroacetic acid (TCA) were purchased from Sigma chemical company. Tetracycline hydrochloride (Himedia), Cycloheximide (Himedia), Ascorbic acid, FeCl_2_.4H_2_O, anhydrous FeCl_3_, K_3_Fe(CN)_6_, anhydrous Na_2_CO_3 _and all other chemicals were of analytical grade.

### Plant Materials

Dragon's blood resin was purchased from wholesale supplier of traditional unani medicine, Ballimaran, Delhi, India, in August 2008, and identified as *Dracaena cinnabari *Balf f. resin. A voucher specimen (NISCAIR/RHMD/Consult/2008-09/1069/100) has been deposited at NISCAIR, New Delhi, India.

### Preparation of extracts

Finely ground samples (200 gm) were extracted sequentially with petroleum ether (PE), dichloromethane (CH_2_Cl_2_) and methanol (MeOH) using a soxhlet assembly for 12 h for each solvent. Extracts were filtered and concentrated under vacuum in a rotary evaporator and stored at 4°C.

### Phytochemical screening of the extracts

#### Determination of the total phenolics content

Total phenolic content of extracts were estimated using a method based on Folin-Ciocalteu reagent [22]. The concentration of total phenolic compounds in different extracts was expressed as mg of gallic acid equivalents (GAE) per gm of dried extract, using a standard curve of gallic acid (concentration range, 0.002-0.01 mg/ml), described by the equation y = 0.0265x (R^2 ^= 0.9977).

#### Estimation of the flavonoids content

Total flavonoid content in the investigated extracts was determined spectrophotometrically [23]. The flavonoids content was expressed as mg of rutin equivalents (RE) per gm of dried extract, by using a standard graph of rutin, covering the concentration in between 0.02-0.2 mg/ml (y = 0.0025x, R^2 ^= 0.9974).

#### Total flavonol content

Total flavonols of extracts were estimated as mg rutin equivalents (RE)/gm extract, from the rutin calibration curve in concentration range, 0.024-0.12 mg/ml (y = 0.0172x, R^2 ^= 0.9979), using a previously reported method [24]. Here, y = absorbance and x = concentration. All experiments were done in triplicate.

### *In vitro *Antioxidant Activities

#### DPPH free radical scavenging activity

Scavenging activity on DPPH was assessed according to the method reported by Blois [25] with a slight modification. Briefly, 100 μl of extracts (0.1-0.5 mg/ml) were mixed with 1 ml of methanolic solution of 0.1 mM DPPH. The mixture was shaken well and incubated at room temperature for 30 min and absorbance was measured at 517 nm in a spectrophotometer. Ascorbic acid, BHT and trolox were used as standards. Experiment was performed in triplicate and averaged. Percent inhibition was calculated from control using the following equation:

$$\text{Scavenging activity }\left(\%\right)=\left(\text{1}-{\text{Absorbance}}_{\text{sample}}{\text{/ Absorbance}}_{\text{control}}\right)\;\times \;100$$

#### ABTS free radical scavenging activity

Trolox equivalent antioxidant capacity (TEAC) was estimated as ABTS radical scavenging activity according to the method of Re et al. [26]. Reagent solution consists of 7 mM ABTS and 2.45 mM potassium persulfate in 100 mM phosphate buffer solution (pH 7.4) and was left to stand for 12 h-16 h at laboratory temperature in the dark to form ABTS radical cation. A working solution was diluted to absorbance values 0.7 ± 0.02 at 734 nm with 100 mM phosphate buffer solution (pH 7.4). 10 μl of standards or resin extracts (2-10 μg/ml) were mixed with the working solution (990 μl) and absorbance was measured at 734 nm after 5 min. Trolox was used as a standard.

$$\text{Scavenging activity }\left(\%\right)=\left(\text{1}-{\text{Absorbance}}_{\text{sample}}{\text{/ Absorbance}}_{\text{control}}\right)\;\times \;100$$

#### Nitric oxide radical scavenging assay

The nitric oxide radical scavenging activity of extracts was determined using the method of Sreejayan and Rao [27]. Sodium nitroprusside in aqueous solution at physiological pH spontaneously generates nitric oxide which interacts with oxygen to produce nitrite ions determined by the Griess reagent. Two millilitre of 10 mM sodium nitroprusside dissolved in 0.5 ml phosphate buffer saline (pH 7.4) was mixed with 0.5 ml of extract at various concentrations (0.05-0.25 mg/ml). The mixture was incubated at 25°C. After 150 min, 0.5 ml of incubation solution was withdrawn and mixed with 0.5 ml of Griess reagent. The mixture was incubated at room temperature for 30 min. The absorbance was measured at 540 nm. The amount of nitric oxide radicals scavenged was calculated following this equation:

$$\text{Scavenging activity }\left(\%\right)=\left(\text{1}-{\text{Absorbance}}_{\text{sample}}{\text{/ Absorbance}}_{\text{control}}\right)\;\times \;100$$

#### Superoxide radical scavenging activity

Superoxide anion scavenging activity was estimated by modified method of Robak and Gryglewski [28]. The reaction mixture consisting of 250 μl of NBT (150 μM), 250 μl of NADH (468 μM) and 250 μl of extract (0.1-0.5 mg/ml) was mixed in sodium phosphate buffer (100 mM, pH 7.4). The reaction was initiated by adding 250 μl of PMS (60 μM) to the mixture. The reaction mixture was incubated at 25°C for 5 min, and the absorbance was measured against the corresponding blank solution. L-Ascorbic acid and trolox were used as positive controls. The superoxide radical scavenging activity was calculated using the following formula:

$$\text{Scavenging activity }\left(\%\right)=\left(\text{1}-{\text{Absorbance}}_{\text{sample}}{\text{/ Absorbance}}_{\text{control}}\right)\;\times \;100$$

#### Fe^2+^- chelating activity

The chelating activity of extract on Fe^2+ ^was measured according to the method of Dinis et al. [29]. 1 ml of extracts (0.1-0.5 mg/ml) was incubated with 50 μl of 2 mM FeCl_2_. The reaction was started by the addition of 200 μl ferrozine (5 mM). After 10 min, the absorbance of ferrous ion-ferrozine complex at 562 nm was read. Na_2_EDTA served as positive control. Triplicate samples were run for each set and averaged. The ability of extracts to chelate ferrous ion was calculated using the following equation:

$$\text{Chelating activity }\left(\%\right)=\left(\text{1}-{\text{Absorbance}}_{\text{sample}}{\text{/ Absorbance}}_{\text{control}}\right)\;\times \;100$$

#### Ferric reducing antioxidant power (FRAP)

The assay was based upon the methodology of Benzie and Strain [30]. The FRAP reagent consisted of 10 mM TPTZ in 40 mM HCl, 20 mM FeCl_3 _and 250 mM sodium acetate buffer (pH 3.6). FRAP reagent was freshly prepared by mixing TPTZ solution, FeCl_3 _solution and acetate buffer in a ratio 1:1:10. A 100 μl of extract solution containing 0.1 mg extracts was mixed with 900 μl of FRAP reagent. After the mixture stood at 37°C for 4 min, the absorbance at 593 nm was determined against blank. Trolox was used as calibration standard in concentration range, 0.002-0.01 mg/ml (y = 0.160x, R^2 ^= 0.981). FRAP values were calculated as mg of Trolox equivalents/gm extract from three determinations and are averaged.

#### Reducing power assay

The reducing power of extracts was determined as per the method of Oyaizu [31]. 1 ml of extracts (0.25-1 mg/ml) was mixed with 2.5 ml of 0.2 M phosphate buffer (pH 6.6) and 2.5 ml K_3_Fe(CN)_6 _(1%). After incubating the mixture at 50°C for 20 min., 2.5 ml of 10% TCA was added, and then mixture was centrifuged at 3000 rpm for 10 min. 2.5 ml of supernatant was mixed with 2.5 ml of distilled water and 0.5 ml FeCl_3 _(0.1%) and the absorbance was measured at 700 nm and compared with standard ascorbic acid.

### Antimicrobial assay

#### Test strains

The antimicrobial activity of the extracts was evaluated on various food borne pathogenic strains obtained from the IMTECH, Chandigarh, India. The antibacterial activity of extracts was screened on Gram-positive bacteria: *Bacillus subtilis *MTCC121 (*Bs*), *Staphylococcus aureus *MTCC96 (*Sa*), *Micrococcus luteus *MTCC106 (*Ml*) and Gram-negative bacteria: *Shigella flexneri *MTCC1457 (*Sf*), *Salmonella enteritidis *MTCC3219 (*Se*), *Proteus mirabilis *MTCC425 (*Pm*), *Enterobacter aerogenes *MTCC2822 (*Ea*), *Escherichia coli *MTCC739 (*Ec*), *Pseudomonas aeruginosa *MTCC2453 (*Pa*). The antifungal activity was tested on *Candida albicans *MTCC227 (*Ca*) and *Aspergillus flavus *MTCC277 (*Af*).

#### Agar disc diffusion method

Antimicrobial activity of extracts was evaluated using agar disc diffusion method [32] against test microorganisms. 100 μl of fresh culture suspension of the test microorganisms was spread on respective media agar plates. The concentration of cultures was 1 × 10^8 ^CFU/ml. For screening, sterile, 6 mm diameter filter paper disc were impregnated with 10 μl of extract equivalent to 0.1 mg of extract after being placed on the surface of the inoculated media agar plates. The plates were stood at 4°C for 2 h before being incubated under optimum conditions for 24 h. Clear inhibition zones around the discs indicated the presence of antimicrobial activity. The diameters of the inhibition zones were measured in millimeter, including the diameter of disc. The controls were set up with equivalent quantities of DMSO as control. Antibiotics tetracycline and cycloheximide were used as reference standards. An inhibition zone of 10 mm or more was considered as high antibacterial activity.

#### Determination of minimal inhibitory concentration (MIC)

Determination of the MIC of extracts against tested microbes was carried out by the broth microdilution method [33]. Extracts were dissolved in DMSO (10% of the final volume) and diluted with culture broth to a concentration of 5 mg/ml. Further 1:2 serial dilutions were performed by addition of culture medium to reach concentrations ranging from 5 to 0.078 mg/ml; 100 μl of each dilution were distributed in 96-well plates. Controls were set up as sterility control (containing culture medium only) and a growth control (containing culture medium plus DMSO). Each test and growth control well was inoculated with 5 μl of a microbial suspension (10^8 ^CFU/ml or 10^5 ^CFU/well). All experiments were performed in triplicate and the microdilution plates were incubated at 37°C for 24 h. For detecting growth, 20 μl of 70% alcoholic solution of INT (0.5 mg/ml) added to each well and incubated further for 30 min, and in those wells, where microbial growth occurred, INT changed from yellow to purple. MIC values were defined as the lowest concentration of each extract, which completely inhibited microbial growth. The results were expressed in milligrams per milliliters.

### *In vitro *nehrotoxicity

#### Kidney cell culture

Porcine proximal tubule cells (LLC-PK1 cell line, National Centre for Cell Science, Pune) were grown until confluency in minimum essential medium (MEM) supplemented with nonessential amino acids and 10% fetal bovine serum without antibiotics, at 37° C in a humidified atmosphere containing 5% CO_2_. The medium was changed every 48 h and the cells were routinely subcultured every 4 d.

#### Cytotoxicity assay

Cells were seeded at a density of 3 × 10^5 ^cells/ml in 96-well plates and allowed to get confluent. Cytotoxicity of the extracts was measured by the MTT assay [34]. MTT (5 mg/ml) was added to the medium in each well, and plates were incubated for 3 h at 37°C. Medium was then removed, and dimethyl sulfoxide (200 μl) was added to each well to solubilize the purple formazan crystals created by mitochondrial dehydrogenase reduction of MTT. After 5 min of additional incubation, absorbance was read at 570 nm on a microplate spectrophotometer. The data were expressed as percent cell viability compared with control (dimethyl sulfoxide).

### Statistical analysis

All experiments were conducted in triplicate (n = 3) and two-way ANOVA (using Graph pad prism 5 statistical software) was used to determine significance of each treatment among standards and extracts. Student's t-test was used to determine the significance between activities at different concentrations of the two extracts. P < 0.0001 vs 0 μg/ml was considered siginificant.

## Results

### Phytochemical screening

In this study, solvents of varying polarity were used to extract and separate the medium and high polarity compounds from the non-polar compounds of the resin. Resin was sequentially extracted with PE, CH_2_Cl_2 _and MeOH using a Soxhlet apparatus. The results of yield, total phenols, total flavonoids and total flavonols of extracts are presented in Table 1. Total phenolic, flavonoid and flavonol content of extracts were in following order: CH_2_Cl_2 _> MeOH > PE; and were significantly different (P < 0.05). Since, total phenolic, flavonoid and flavonol content of PE extract was very less, this extract was not further taken for antioxidant assays.

**Table 1:** Yield and phytochemical content of the extracts from Dragon's blood resin

Extracts	Yield (%)	Total phenols (mg GAE/g extract)	Total flavonoids (mg RE/g extract)	Total flavonols (mg RE/g extract)
PE	0.556 ± 0.01	0.704 ± 0.246	Tr	tr
CH_2_Cl_2_	2.605 ± 0.168	263.019 ± 3.717	229.333 ± 10.066	16.86 ± 0.581
MeOH	53.922 ± 0.468	177.358 ± 6.178	82.667 ± 8.327	56.395 ± 1.744

tr, trace (<0.05).

### Antioxidant activity

The antioxidant effects of plant products must be evaluated by combining two or more different *in vitro *assays to get relevant data, because of the complex nature of phytochemicals. Each of these tests is based on one feature of the antioxidant activity, such as the ability to scavenge free radicals, or the metal ion chelation.

#### DPPH free radical scavenging

**Figure 1:**
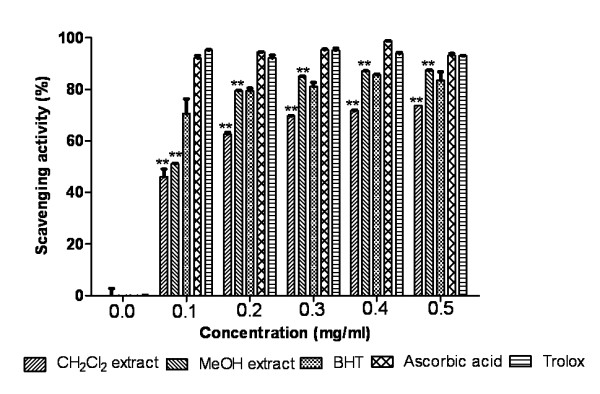
**DPPH scavenging activity**. Effect of Dragon's blood resin extracts and standards BHT, ascorbic acid, trolox on DPPH radical scavenging study. The data is expressed as% scavenging of DPPH radicals. **P < 0.0001 vs 0 μg/ml.

As depicted in Figure 1, CH_2_Cl_2 _extract and MeOH extract showed steady increase in percent inhibition of the DPPH radicals with concentration. MeOH extract showed higher DPPH scavenging activity than standard BHT but less than ascorbic acid and trolox. CH_2_Cl_2 _and MeOH extracts of resin were able to inhibit the formation of DPPH radicals with an IC_50_, 0.1357 ± 0.0069 mg/ml and 0.0942 ± 0.0024 mg/ml respectively. The extracts and standards were significantly different (P < 0.0001).

#### ABTS free radical scavenging

**Figure 2:**
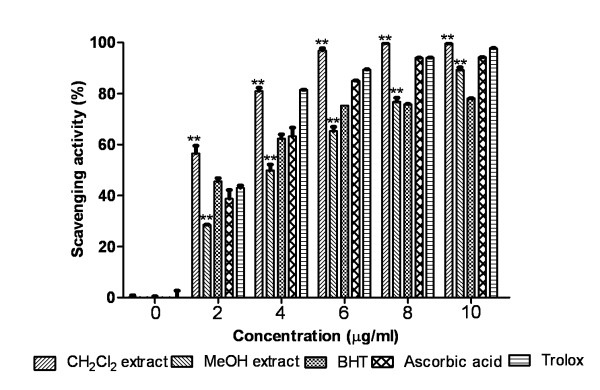
**Total antioxidant activity**. Effect of Dragon's blood resin extracts and standards BHT, ascorbic acid, trolox on ABTS radical cation decolorization assay. The percentage of inhibition was plotted against concentration of samples. **P < 0.0001 vs 0 μg/ml.

The results are demonstrated in Figure 2 and showed that CH_2_Cl_2 _extract (IC_50_= 0.0018 ± 0.0001 mg/ml) was more active than MeOH extract (IC_50 _= 0.0041 ± 0.0002 mg/ml) and standards BHT, trolox and ascorbic acid. The two extracts of the resin and standards showed significant difference (P < 0.0001) in ABTS scavenging activity.

#### Nitric oxide radical scavenging assay

**Figure 3:**
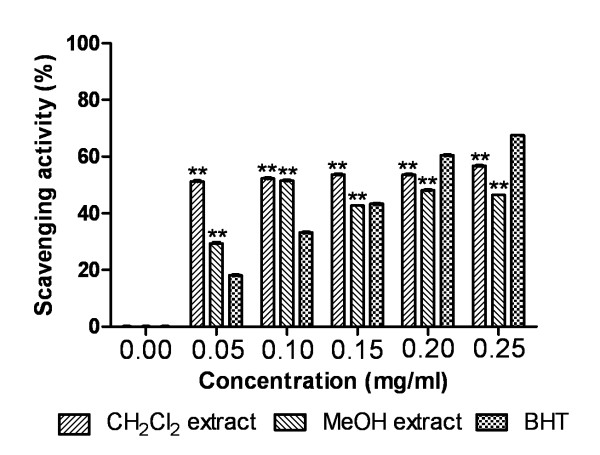
**Nitric oxide radical scavenging activity**. Effect of Dragon's blood resin extracts and standard BHT on nitric oxide scavenging assay. The percentage of scavenging was plotted against concentration of samples. **P < 0.0001 vs 0 μg/ml.

The percent inhibition of nitric oxide radicals was shown in Figure 3. The results show that CH_2_Cl_2 _extract had significantly higher scavenging activity (53.675 ± 0.111%) than standard BHT (43.341 ± 0.027%) at 0.15 mg/ml. MeOH extract was inconsistent in scavenging nitric oxide radicals with increase in concentration and found to show 51.51 ± 0.146% scavenging at 0.1 mg/ml. The extracts and standard were found to be significantly different (P < 0.0001).

#### Superoxide radical scavenging assay

**Figure 4:**
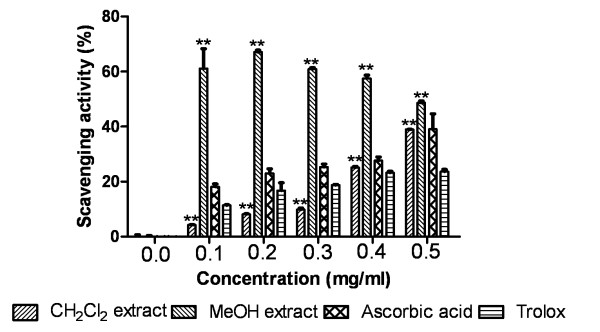
**Superoxide radical scavenging activity**. Effect of Dragon's blood resin extracts and standards ascorbic acid, trolox on superoxide scavenging assay. The percentage of scavenging was plotted against concentration of samples. **P < 0.0001 vs 0 μg/ml.

As demonstrated in Figure 4, MeOH extract showed significantly higher superoxide radical scavenging activity than CH_2_Cl_2 _extract, ascorbic acid and trolox. There was significant difference in superoxide radical scavenging activity of extracts and standards (P < 0.0001).

#### Iron (II) chelating activity

**Figure 5:**
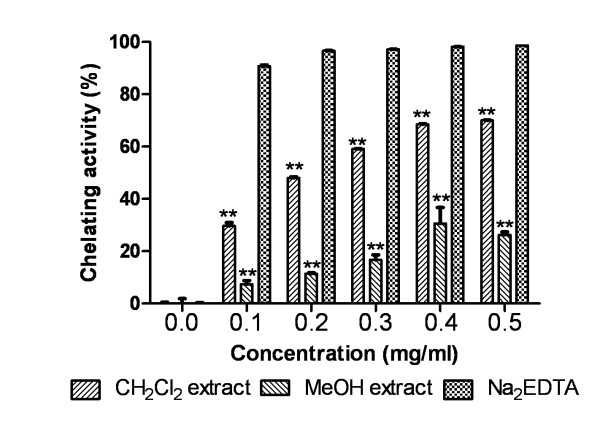
**Metal chelating activity**. Effect of Dragon's blood resin extracts and standard Na_2_EDTA on metal chelating assay. The percentage of chelation was plotted against concentration of sample. **P < 0.0001 vs 0 μg/ml.

As demonstrated in Figure 5, CH_2_Cl_2 _extract showed metal chelating activity with an IC_50 _value of 0.2195 ± 0.0052 mg/ml and was less potent chelator than standard Na_2_EDTA. MeOH extract was found to be very less reactive showing a maximum of 25.96% inhibition at 0.5 mg/ml. The two extracts and standard were significantly different in metal chelation (P < 0.0001).

#### FRAP activity

In this assay, reduction of the ferric-tripyridyltriazine to the ferrous complex forms an intense blue color which can be measured at a wavelength of 593 nm. The intensity of the color is related to the amount of antioxidant reductants in the samples. The ferric reducing activity of CH_2_Cl_2 _and MeOH extracts of resin were found to be 2.831 ± 0.598 and 1.366 ± 0.017 mg trolox equivalents/gm extract respectively. There was significant difference in FRAP values of the two extracts with P < 0.05.

#### Reducing power assay

**Figure 6:**
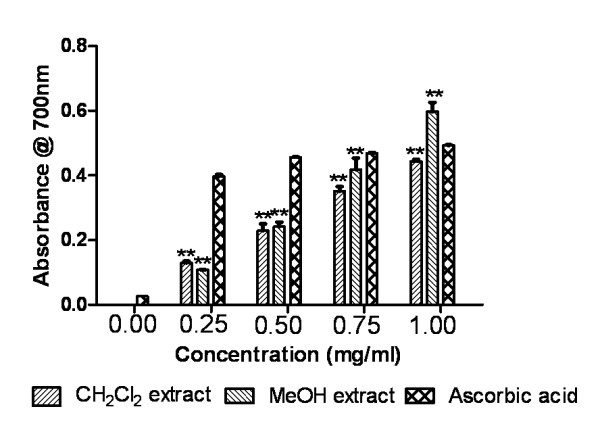
**Reducing power assay**. Reducing power activities of Dragon's blood resin extracts and standard ascorbic acid. The absorbance (A_700_) was plotted against concentration of sample. **P < 0.0001 vs 0 μg/ml.

Figure 6 depicts the reducing power activity of extracts were comparable to that of standard ascorbic acid. Reducing ability increased well with amount of extracts. Extracts and standard were significantly different (P < 0.0001).

#### Correlation between the total phenolic contents with the antioxidant activity

**Figure 7:**
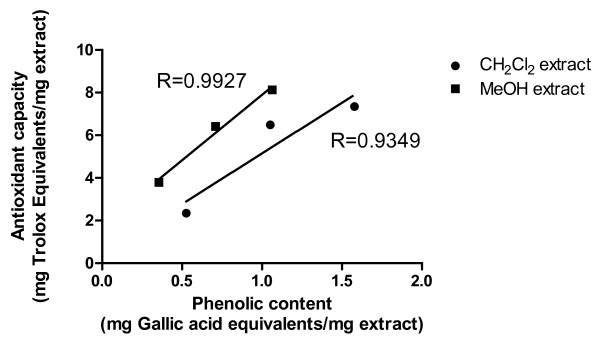
**Correlation of antioxidant activity with phenolic contents**. The relationship between total phenolic content in individual extract and their antioxidant capacity. The correlation analyses were described as linear correlation coefficient (R).

As showed in Figure 7, the total phenolic content of MeOH extract significantly correlated with total antioxidant activity (R = 0.9927), whereas the correlation coefficients for CH_2_Cl_2 _extract was found to be greater than 0.9 (R = 0.9349) which proved that the phenolic contents of these extracts highly attributed their antioxidant activity.

### Antimicrobial activity

In this study, nine different bacterial and two fungal species were used to screen the possible antimicrobial activities of Dragon's blood resin extracts. Antimicrobial activity assay against these microbial strains showed that CH_2_Cl_2 _extract was most active amongst extracts, as shown in Table 2. PE extract exhibited weak antibacterial and antifungal activities (data not shown here). While, CH_2_Cl_2 _and MeOH extracts displayed activity against all test bacteria except *S. enteritidis*. It is remarkable to note that CH_2_Cl_2 _extract was also active against fungi *C. albicans *and *A. flavus*.

**Table 2:** Antimicrobial activity of the extracts

Microorganisms	Zone of inhibition (mm)				MIC (mg/ml)	
	
	**CH**_**2**_**Cl**_**2 **_**extract**	MeOH extract	Tet	CyX	**CH**_**2**_**Cl**_**2 **_**extract**	MeOH extract
*Bs*	11.33 ± 0.58	10.33 ± 0.58	11.0 ± 0.0	ND	0.156	0.625
*Ml*	17.67 ± 0.58	17.33 ± 0.58	35.0 ± 0.0	ND	0.156	1.25
*Sa*	9.0 ± 0.0	10.33 ± 0.58	16.0 ± 0.0	ND	1.25	1.25
*Sf*	14.0 ± 0.0	10.33 ± 0.58	15.0 ± 0.0	ND	0.156	1.25
*Pm*	11.33 ± 0.58	12.0 ± 1.0	16.0 ± 1.0	ND	0.3125	1.25
*Se*	NA	NA	NA	ND	>5	>5
*Ea*	8.67 ± 0.58	9.67 ± 0.58	10.0 ± 0.0	ND	0.625	1.25
*Ec*	9.0 ± 0.0	9.0 ± 0.0	11.67 ± 0.58	ND	0.3125	0.3125
*Pa*	7.67 ± 0.58	8.0 ± 0.0	NA	ND	>5	>5
*Ca*	8.0 ± 0.0	NA	ND	NA	>5	>5
*Af*	9.0 ± 0.0	NA	ND	14.0 ± 3.61	>5	>5

NA, No antimicrobial activity. ND, Not determined, Tet, Tetracycline, CyX, Cycloheximide, Inhibition zones including the disc diameter (6 mm); <8.0 mm, weak activity; 8.0-9.0 mm, slight low activity; 9.1-10.0 mm, middle activity; 10.1-11.0 mm, slight high activity; 11.1-12.0 mm, moderate high activity; over 12.0 mm, very high activity.

### *In vitro *nephrotoxicity

**Figure 8:**
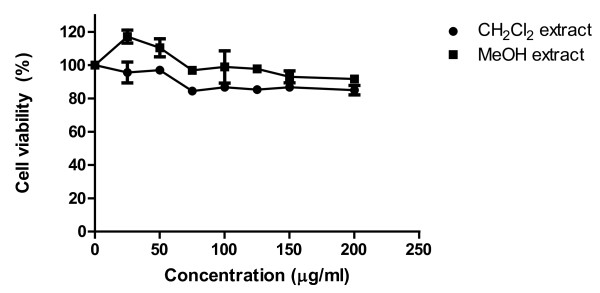
**Cytotoxicity of Dragon's blood resin extracts**. LLC-PK1 cells were treated with various concentrations of extracts to determine percent cell viability through MTT assay.

Cytotoxicity of resin extracts to LLC-PK1 cells was measured by MTT assay. As depicted in Figure 8, CH_2_Cl_2 _extract showed more than 80% viability of cells at concentrations ≤200 μg/ml. Cell viability was not also significantly altered by MeOH extract, at up to 200 μg/ml. These results suggest that concentrations of extracts were not toxic to LLC-PK1 cells below 200 μg/ml.

## Discussion

Food preservation is one of the oldest technologies used by human beings to avoid its spoilage. Food preservation by definition is a process by which certain foods like fruits and vegetables are prevented from getting spoilt for a long period of time. The color, taste and nutritive value of the food are also preserved. Besides damage to living cells, free radicals are the major cause of food deterioration through lipid oxidation, which ultimately affects the taste and smell of foods.

It is well known that phenolic compounds possess good antioxidant properties [35,36]. Phenolic compounds are secondary metabolites of plants and can act as antioxidants by many potential pathways such as free radical-scavenging, oxygen radical absorbance, and chelation of metal ions [37]. Flavonoids belong to phenolic compounds and are excellent antioxidant compounds because they are highly reactive as hydrogen and electron donor [38-41]. The mode of the actions of flavonoids is through scavenging or chelating processes [42,43]. CH_2_Cl_2 _and MeOH extracts of Dragon's blood resin were found to show high content of phenolic compounds and good antioxidant activity. DPPH assay demonstrated that both CH_2_Cl_2 _and MeOH extracts were potent in scavenging DPPH free radicals, suggesting the presence of compounds capable of donating hydrogen to the free radicals. In ABTS radical scavenging assay, CH_2_Cl_2 _extract showed significantly higher scavenging as compared to standards BHT, trolox and ascorbic acid; which implies that this extract could be a useful candidate for food preservation as well as for treatment of radical related pathological damage. Nitric oxide radicals play an important role in pathogenesis of inflammation and their toxicity multiplies when they form peroxynitrite after reacting with O_2_^•^^- ^radicals, damaging biomolecules like proteins, lipids and nucleic acids [44]. The level of nitric oxide was significantly reduced in this study by the CH_2_Cl_2 _extract and was higher than BHT at 0.15 mg/ml. These results may explain the use of Dragon's blood resin for the treatment of inflammation and for wound healing. Superoxide anion radical is supposed to be one of the strongest reactive oxygen species among the free radicals. The scavenging of these radicals by the MeOH extract as compared to standards ascorbic acid and trolox, suggested that it is a potent scavenger of superoxide radicals.

The transition metal ion Fe^2+ ^possesses the ability to move single electrons thus allowing the formation and propagation of many radical reactions, even starting with relatively nonreactive radicals [45]. The mechanism to avoid reactive oxygen species formation, associated with redox active metal catalysis is through chelation of the metal ions. In metal chelating assay, presence of chelating agents disrupts the formation of ferrozine-Fe^+2 ^complex monitored by measuring the decrease in the red color of the complex at 562 nm and thus, estimating the chelating activity of the coexisting chelator. CH_2_Cl_2 _extract was found to be better metal chelator than MeOH extract.

The reducing capacity of a compound may serve as a significant indicator of its potential antioxidant activity. Fe^+3 ^reduction, an indicator of electron donating activity, is considered to be an important mechanism of antioxidant activity of phenolics [46]. In the reducing power assay, the antioxidants in the samples reduce Fe^3+ ^to Fe^2+ ^by donating an electron. Amount of Fe^2+ ^complex can then be measured spectrophotometrically at 700 nm. Increasing absorbance at 700 nm indicates an increase in the reductive ability. Both extracts displayed increasing reducing power in dose dependent manner. Since, MeOH extract showed higher reducing power than ascorbic acid at 1 mg/ml, this extract must be richer in compounds capable of donating hydrogen atom.

Correlation studies of extracts revealed strong correlation between total phenolic content and antioxidant activity of the extracts. Antioxidant activity of the two extracts of resin can be the result of their high phenolic content, as phenolics have been reported to act as reducing agents, hydrogen donors, free radical quenchers and metal chelators.

Apart from oxidative damage, contamination by microbes is another common problem during food preservation and also possesses serious health threats to those consuming it. Antimicrobial activity assay against different food borne pathogens showed that CH_2_Cl_2 _extract was most active amongst extracts, as shown in Table 2. Of the species used, *S. aureus *is the most common cause for food poisoning. *B. subtilis *is a common cause of food spoilage, mainly in canned foods. *M. luteus *has also been reported to be a food contaminant. In developing countries, *S. flexneri *is most predominant cause for acute bloody diarrhea known as bacillary dysentery. *P. mirabilis *has also been associated with food spoilage in fresh meat, poultry and seafood. *S. enteritidis *has become single most common cause of food poisoning causing gastroenteritis. *E. coli*, *E. aerogenes *and *P. aeruginosa *also causes food spoilage. CH_2_Cl_2 _extract showed strong antimicrobial activity with MIC values ranging from 0.156-1.25 mg/ml against test microorganisms and could be attributed to its high phenolic content. MeOH extract also exhibited good inhibition to test pathogens as MIC values were in range of 0.3125-1.25 mg/ml. Two extracts did not demonstrate inhibition to *P. aeruginosa *and *S. enteritidis*, while *M. luteus *was most sensitive to the extracts. Two extracts were also tested against fungal food pathogens *C. albicans *and *A. flavus*; only CH_2_Cl_2 _extract was able to inhibit fungal pathogens. Thus, the two extracts possess significant inhibitory activity to be used as antibacterial agent.

Exposure to drugs and chemicals often results in toxicity to living organisms and are not equally toxic to all parts of a living system because the toxic actions of many compounds are manifested in specific organs. Many organs, including the kidney, are capable of metabolizing chemicals to toxic reactive intermediates. The increasing use of *in vitro *techniques using isolated renal cells, nephron fragments, or cell cultures derived from specific renal cell types has helped in studying nephrotoxicity. Kidney derived cells are cultured in presence of test compounds whose cytotoxicity is then determined and serves as an indicator of potential nephrotoxicity. LLC-PK1 is an established cell line derived from normal porcine kidneys that has been widely used to study nephrotoxicity of a number of compounds [47,48]. Cytotoxicity studies showed that CH_2_Cl_2 _extract and MeOH extract were not toxic to LLC-PK1 cells upto the concentration of 200 μg/ml. Thus, resin extracts can be used as a source of food preservative at a concentration below 200 μg/ml without showing any nephrotoxicity.

## Conclusions

Present study revealed that Dragon's blood resin extracts demonstrated high phenolic content and potent antioxidant activity, achieved by free radical scavenging, FRAP, metal chelating and reducing power assays. The CH_2_Cl_2 _extract had the highest radical-scavenging activity, metal chelating activity and highest total phenolic content among all extracts. CH_2_Cl_2 _extract also exhibited good inhibitory activity against bacterial and fungal food pathogens. Thus, CH_2_Cl_2 _extract of Dragon's blood resin could be considered as possible source of food preservative. The wide use of this resin in traditional medicinal systems as anti-inflammatory, wound healing agent and against diarrhea, dysentery may be in part due to its antioxidant and antimicrobial properties. Further activity guided isolation, characterization and toxicity of the extract is in progress to identify the full composition of the extract and the exact compound(s) responsible for its bioactivity, to be developed as food preservative agents.

## Competing interests

The authors declare that they have no competing interests.

## Authors' contributions

DG carried out the study design, specimen collection, experimental work, data collection and interpretation, literature search and manuscript preparation. RKG supervised the work, evaluated the data and corrected the manuscript for publication. All authors have read and approved the final manuscript.

## Pre-publication history

The pre-publication history for this paper can be accessed here:

http://www.biomedcentral.com/1472-6882/11/13/prepub
